# Incidental venous thrombosis in oncology in a sub-Saharan tertiary hospital

**DOI:** 10.3332/ecancer.2024.1793

**Published:** 2024-11-07

**Authors:** Etienne Okobalemba Atenguena, Joseph Francis Nwatsock, Berthe Sabine Esson Mapoko, Lionel Fossa Tabola, Kenn Chi Ndi, Jérôme Boombhi, Paul Ndom

**Affiliations:** 1 Medical Oncology, General Hospital, PO Box 5408, Yaoundé, Cameroon; 2 Radiology, General Hospital, PO Box 5408, Yaoundé, Cameroon; 3 Haematology Oncology, Central Hospital, PO Box 87, Cameroon; 4 Cardiology, General Hospital, PO Box 5408, Yaoundé, Cameroon

**Keywords:** thrombosis, incidental, oncology, Cameroon

## Abstract

The relationship between cancer and thrombosis was initially highlighted in the 19th century. Vascular complications in oncology can be arterial or venous thrombosis, and incidental pulmonary embolism is a growing challenge. We aimed to describe the frequency and clinical characteristics of cancer patients with incidental venous thromboembolism (iVTE). We conducted a descriptive study at the Yaounde General Hospital. We included patients with a confirmed diagnosis of cancer, followed up on an outpatient basis, in whom an iVTE was identified on a computed tomography scan performed to evaluate tumour status over a 6-month period. Of the 359 patients, 19 had venous thromboses, representing a frequency of 5.3%. The mean age was 51.2 years. The sex ratio was 1.1 in favour of males. Comorbidities found were diabetes, hypertension and obesity. Colon cancer (5), ovarian cancer (3) and lung cancer (3) were the most frequent diagnoses. All patients had advanced disease with 14 (73.7%) being naive to anticancer treatment. Pulmonary arteries were the most affected vessel (63.1%). The frequency of iVTE in a sub-Saharan context was around 5%.

## Background

The relationship between cancer and thrombosis was first demonstrated in the 19th century, with several studies following, in a bid to better understand the different mechanisms involved [1]. Components of Virchow’s triad (blood stasis, hypercoagulability and endothelial damage) are generally found in patients with cancer, predisposing them to a greater risk of thrombosis. Vascular complications in oncology can be arterial or venous thrombosis [2]. Of these two types, venous thromboembolism (VTE) is the most common and includes deep vein thrombosis and pulmonary embolism (PE). It can occur at any time during the follow-up of cancer and can sometimes reveal the disease [3]. Patients with cancer have a 5 to 7 times greater risk of developing VTE [4]. In cancer patients, VTE is associated with a worse prognosis and increased medical costs [5, 6]. These varying vascular events can be identified on a contrast-enhanced computed tomography (CT) scan. People with cancer often undergo multiple routine staging and response to treatment

CT examinations [7]. Advances in medical imaging studies, including CT, have allowed for improved visualisation of the pulmonary arterial tree, which has led to an increase in the detection of PE as an incidental finding [8, 9]. In low- and middle-income countries, as is the case in most of sub-Saharan Africa, the availability and cost of CT scans are obstacles to their use. There is less than one CT scan per one million population for low- and middle-income countries, compared to 40 scans per one million population for high-income countries [10]. In the literature, few studies on thrombosis in patients with cancer have been published in sub-Saharan Africa. Virtually all of the epidemiological data on VTE come from high-income countries [11]. A study on the rates of VTE in high-income, upper middle-income and lower middle/low-income countries concluded that the rates of VTE are substantially higher in high-income than in low-income countries [12]. We sought to describe incidental venous thromboembolism (iVTE) found on CT in outpatients with cancer in a sub-Saharan African country to obtain data on the subject. These data, which will make it possible to assess the efficiency of iVTE detection, will remind clinicians to treat these patients effectively. This study aimed to describe the frequency and clinical characteristics of cancer patients with iVTE.

## Methods

We carried out a single-centre descriptive cross-sectional study at the Yaounde General Hospital, the referral hospital for the management of cancer in the city of Yaounde, the capital of Cameroon, a country in Central Africa. This study was conducted from January to June 2022. The study population consisted of consenting outpatients with pathologically confirmed cancer in whom a staging contrast-enhanced thoraco-abdomino-pelvic CT scan incidentally found VTE. iVTE was defined as a filling defect on contrast-enhanced CT scans performed for reasons other than clinical suspicion of VTE. In this study, iVTE was classified into two categories depending on whether the thrombosis occurred in or outside the thorax: incidental pulmonary embolism (iPE) and unusual deep VTE. iPE was defined as a filling defect in one or more pulmonary arteries in a contrast-enhanced chest CT. We defined unusual deep site VTE as thrombosis that affects any vein located either in the abdomen or in the pelvis.

Thoraco-abdomino-pelvic CT scans were all requested and performed in the same hospital, which has three radiologists who each have more than 5 years of experience. The CT scan used realises slices of 1 mm thickness. Our sampling was consecutive and non-probabilistic. All new and old patients who visited the oncology outpatient unit during the study period were approached by the investigator to check for study eligibility, and signed informed consent forms were obtained.

A pre-established questionnaire was used to collect the data. We collected the different variables on a handwritten questionnaire before entering them into the analysis software.

The variables collected from patient files included patient demographics, past history, cancer characteristics, thrombosis diagnostic imaging data and initial VTE management. Demographic data included age and sex, and past history included the existence of comorbidities, the nature of these comorbidities and a history of COVID-19 infection. Cancer characteristics included primary site, histologic type and American Joint Committee on Cancer stage.

Thrombosis diagnostic imaging data included the reason for imaging and the site of thrombosis. The reason for imaging was one of the following: initial evaluation if the patient had not yet received any anticancer treatment, midterm evaluation if the patient had received some systemic anticancer treatment and surveillance if the patient had completed anticancer treatment. Radiology reports were reviewed to determine the location of the VTE.

The incidence rate was calculated by dividing the number of iVTE cases by the number of thoraco-abdomino-pelvic CT scans performed during a 6-months period.

Statistical analysis was performed using the Epi Info version 5 software. For continuous data, the number of subjects, average, standard deviation (SD), median, minimum value and maximum value are presented. For categorical data, the frequency and percentage are displayed, and the number of cases are shown for categories collected multiple times. The descriptive statistics (average, SD, median, minimum value, maximum value and percentage) are rounded up and presented to one decimal point.

We obtained a research authorization from the administration of the General Hospital of Yaounde. Free and informed consent was obtained from each patient prior to participation in the study.

## Results

During our study period, we recruited 19 patients with incidental venous thrombosis among the 359 who underwent routine staging or treatment assessment CT scans. This represents a frequency of 5.3%.

### Patient characteristics

The average age was 51.2 years. The sex ratio was 1.1 in favour of males. Of our patients, six had a comorbidity. Hypertension was the most frequent (*n* = 3) comorbidity. Three patients were found to have had a previous COVID-19 infection, (Table 1).

### Disease characteristics

The largest number of incidental VTE were seen in patients with colon cancer (*n* = 5), followed by ovarian cancer (*n* = 3) and lung cancer (*n* = 3). Adenocarcinoma was the most frequent histological type (*n* = 13), followed by squamous cell carcinoma (*n* = 4). All 19 patients had advanced cancer: 5 had stage III cancer and the other 14 had stage IV cancer (Table 2).

### Imaging indication

Fourteen patients underwent imaging for an initial evaluation of their cancer, four were at the mid-term evaluation. Following the diagnosis of VTE, 13 individuals (68.4%) were initially treated with an oral anticoagulant (rivaroxaban). The remaining six patients did not receive any anticoagulant treatment (Table 3).

### Anatomical location of thrombosis

Pulmonary arteries were the most affected vessel (63.1%). We had 47.4% of deep VTE occurring outside the thorax. Some patients had multiple affected vessels (Table 4).

## Discussion

In our study, the frequency of iVTE was 5.3%. The rate of iVTE varies between studies, ranging from 1.4% to 9% [13, 14]. The wide variation in the incidence of iVTE, particularly iPEs can be explained by the high heterogeneity between studies. The slice thickness of the CT scans could also be a possible explanation. Dentali *et al* [14] reported a higher incidence of iPE when using CT scans with <5 mm slice thickness compared to >5 mm slice thickness (3.0% versus 2.0%). In our study, the thickness of the CT slices was less than 5 mm, which probably enabled us to obtain incidence rates closer to the high values found in the literature.

Of the 19 patients with iVTE, 10 were male. The role of gender in the occurrence of thrombosis varies between studies. Women are more likely to develop VTE, whereas men are more likely to develop arterial thrombosis [15, 16].

Several risk factors for iVTE have been described in the literature. We looked for different risk factors in patients in this study. Diabetes, hypertension and obesity were the comorbidities found in our patients. Among our patients, 2 (10.5%) were obese. Obesity is associated with a high risk of ischemic stroke, as well as VTE in patients of both sexes [17, 18]. In their study, Siegal *et al* [12] who worked on the effect of country income level on VTE risk, found that the effect of country income level on VTE risk was markedly stronger in people with a lower BMI compared to those with higher BMI (*p* < 0·001). In our sample, the majority of patients had a BMI of less than 30 (17/19). They would therefore be deemed to be at less risk of VTE.

Three of our patients had a COVID-19 infection in the past. After a respiratory or other infection, there is a two- to three-fold increased risk of VTE [19]. This risk decreases after infection resolution. However, this risk may persist for a year or more [19]. The risk of VTE after COVID-19 infection is mixed. A meta-analysis in patients with a previous severe infection in the first wave found a 13% incidence of VTE [20]. Another study including a control group did not find a higher risk in patients with a history of COVID-19 infection [21].

In our sample, the most common existing malignancy was colon cancer; followed by ovarian and lung cancers. The incidence of venous thrombosis differs according to the primary site of the cancer. It is higher in primary sites such as the liver, pancreas (about 4%) and ovary, compared to bladder or head and neck cancers (1%) [22, 23].

Adenocarcinoma was the most frequent histological type in our population. The histological type of cancer is related to the risk of VTE. The risk is higher for adenocarcinoma than for squamous cell carcinoma in patients with bronchial cancer [24]. Some studies suggest a higher frequency of VTE in patients with bronchial, pancreatic or gastrointestinal adenocarcinoma secreting mucin [25]. However, there was no significant difference between the different histological types of breast or colon cancers [26, 27].

All patients had advanced-stage cancer in our sample. Patients with advanced cancer would be at a higher risk of developing VTE [28]. Patients with metastatic disease had a 1.5-fold higher incidence of PE compared to patients without metastases [29].

Thrombosis was diagnosed in 15 patients (75%) at the time of initial evaluation. The period immediately following the diagnosis of cancer is a period in which the risk of developing VTE is high [30]. Of our patients, 4 (21.1%) had already started chemotherapy. Chemotherapy is an important risk factor for VTE in patients with cancer. Patients undergoing treatment have a 6- to 7-fold greater risk of developing VTE, and within 12 months of chemotherapy initiation, the rate of VTE is higher in patients with cancer [31]. Surgery, radiotherapy, hospitalisation, central venous catheters and other anti-cancer drugs [15, 32, 33] are likewise, risk factors for VTE. The mechanisms explaining treatment-related thrombosis are not fully understood. Drugs may activate and disrupt the integrity of the endothelium, decrease anticoagulants and increase pro-coagulants such as tissue factor leading to activation of coagulation or directly or indirectly activating platelets. It is difficult to differentiate between the pro-thrombotic actions of the tumour itself and those of the various anti-cancer treatments administered to patients.

Of our patients, 26.3% had central iPE. The prevalence of central PE in incidental PE cases diagnosed varied from 23% to 63.6% in the literature [34, 35]. This infers that in many cancer patients, continuous prothrombotic states due to cancer contribute to the gradual expansion of pulmonary thrombosis resulting in hemodynamic adaptations in patients.

Some patients had unusual deep VTE, e.g., vena cava thrombosis. Generally, the signs and symptoms of superior vena cava obstruction are progressive. Isolated intravascular thrombosis is rare [36]. Cancers are the cause in 2/3 of cases. Occlusion is due to thrombosis in 1/3 of cases, reflecting the increased use of intravascular devices such as central venous catheters, catheter portals and pacemakers [37]. None of our patients had an intravascular device. Inferior vena cava thrombosis is rare [38]. Although inferior vena cava thrombosis is not frequently detected, it can be associated with complications such as post-thrombotic syndrome (90%), venous claudication (45%), PE (30%) and venous ulceration (15%) [39].

Of our patients, 13 (68%) received treatment for the iVTE. Those 13 patients all initially received rivaroxaban. Qdaisat *et al* [35] showed that cancer patients with iPE have a worse prognosis than those without PE. They argued that patients with IPE should be treated with proper management plans similar to their symptomatic counterparts [35].

This study on incidental venous thrombosis in oncology in a sub-Saharan tertiary hospital proves that this is a real clinical situation. Medical teams caring for cancer patients in sub-Saharan Africa must pay close attention to the CT scans performed in order to optimally manage any iVTE. This proper management will help to avoid potentially fatal acute complications.

## Limitations

The present study has several limitations owing to its retrospective and single-centre design. The small sample size may limit the applicability of our findings. We did not collect respiratory symptoms data. Some patients may have had symptoms when questioned further, possibly increasing symptomatic VTE, and then decreasing the percentage of incidental VTE. Radiology reports were used without reviewing the CT images and iVTE may have been missed in initial reports.

## Conclusions

We found that the incidence rate of incidental VTE in a sub-Saharan context is around 5%. iVTE was most frequently observed in patients with colon, ovarian and lung cancer. iPE was more frequent than unusual deep VTE. Policy makers in sub-Saharan Africa should take actions like: set up programmes to make CT scans accessible, and make available recommendations for the management of iVTE or nothing will change. They could consider oral anticoagulants, which solve a number of problems (few drug interactions, no need for biological monitoring).

## Conflicts of interest

All authors have no conflict of interest relevant to this study to declare.

## Funding

This study was self-funded.

## Figures and Tables

**Figure 1.**
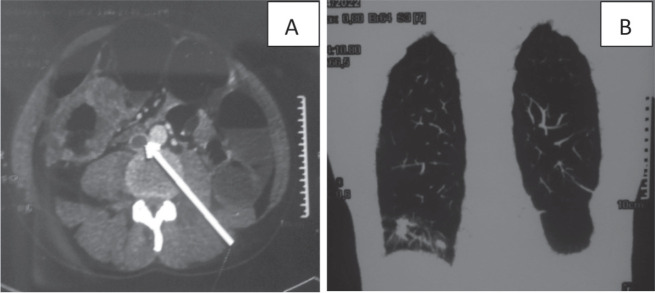
CT scan of a 37-year-old man: (a): Cross-section of inferior vena cava thrombosis (white arrow) and a circumferential mass of the right colon. (b): Frontal section of lung metastases.

**Figure 2.**
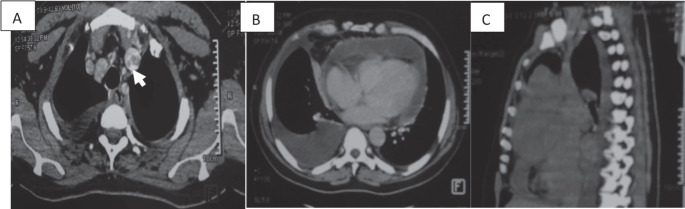
CT scan of a 58-year-old man (a): Cross-section of brachiocephalic trunk thrombosis (white arrow). (b): Cross-section of right pericardial and pleural effusion. (c): Sagittal section with a mediastinal mass.

**Table 1.** Patient characteristics.

Patient characteristics	*N* = 19
Mean age (range)	51.6 (33–75) years
Gender	
Male	10
Female	9
Presence of comorbidities	
Yes	6
No	13
Type of comorbidities^a^	
Hypertension	3
Diabetes mellitus	2
Obesity	2
History of COVID-19 infection	
Yes	3
No	16

a Some patients had several comorbidities

**Table 2.** Cancer characteristics.

Cancer characteristics	*N* = 19
Primary site	
Colon	5
Ovary	3
Lung	3
Head and neck	2
Stomach	2
Oesophagus	2
Uterine cervix	1
Muscles	1
Histological subtypes	
Adenocarcinoma	13
Squamous cell	4
Lymphoma	1
Sarcoma	1
Stage	
III	5
IV	14

**Table 3.** Imaging indication.

Items	*N* = 19
Indication	
Initial evaluation	14
Mid-term evaluation	4
Surveillance	1
Initial therapy	
Oral anticoagulation (rivaroxaban)	13
No initial treatment	6

**Table 4.** Anatomical location.

Type of thrombosis	Affected vessel^a^	*N* = 19	Frequency
PE	Peripheral	7	36.8%
	Central	5	26.3%
Unusual deep VTE	Renal vein thrombosis	4	21.1%
	Inferior Vena Cava Tumour Thrombosis^b^	3	15.8%
	Superior Vena Cava Tumour Thrombosis^c^	2	10.5%

a A patient can have multiple affected vessels

b See figure 1

c See figure 2
